# Supplementary material for: Versican binds collagen via its G3 domain and regulates the organization and mechanics of collagenous matrices
Source: J Biol Chem. 2024 Nov 5;300(12):107968. doi: 10.1016/j.jbc.2024.107968 (PMC11626796; doi:10.1016/j.jbc.2024.107968)
Supplement: Supporting information [file mmc1.docx]

**Supporting Information**

**Versican binds collagen via its G3 domain and regulates the organization and mechanics of collagenous matrices**

Dongning Chen^1,2,3^, Yu Du^3,4^, Jessica Llewellyn^3,4^, Arkadiusz Bonna^5^, Biao Zuo^6^, Paul A. Janmey^1,2,3,7,8^, Richard W. Farndale^5^, Rebecca G. Wells^1,2,3,4^

^1^Department of Bioengineering, University of Pennsylvania, Philadelphia, PA 19104

^2^The Materials Research Science & Engineering Center, University of Pennsylvania, Philadelphia, PA 19104

^3^National Science Foundation Center for Engineering MechanoBiology, Philadelphia, PA 19104

^4^Department of Medicine, University of Pennsylvania, Philadelphia, PA 19104

^5^Triple Helical Peptides Ltd, Cambridge CB22 5DU, UK

^6^Electron Microscopy Resource Laboratory, Department of Biochemistry & Biophysics, University of Pennsylvania, Philadelphia, PA 19104

^7^Institute for Medicine and Engineering, University of Pennsylvania, Philadelphia, PA 19104

^8^Department of Physiology, University of Pennsylvania, Philadelphia, PA 19104


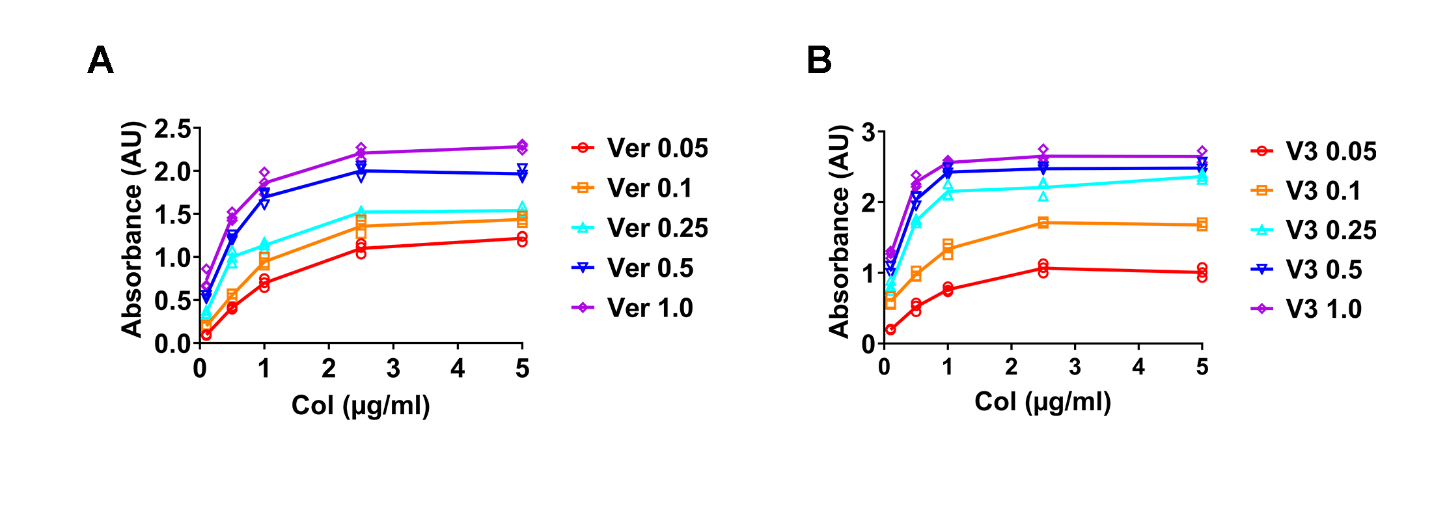


**Figure S1.** Versican-collagen binding is dose-dependent. **A, B)** The interaction between collagen and versican (A) or V3 (B) at increasing collagen and versican concentrations. A 96-well plate was coated with versican or V3 at 0.05, 0.1, 0.25, 0.5 and 1.0 µg/ml and collagen was added at 0.1, 0.5, 1.0, 2.5 and 5.0 µg/ml. The absorbance values represent the amount of collagen bound to versican or V3. Three independent experiments were carried out for each; all data were shown.


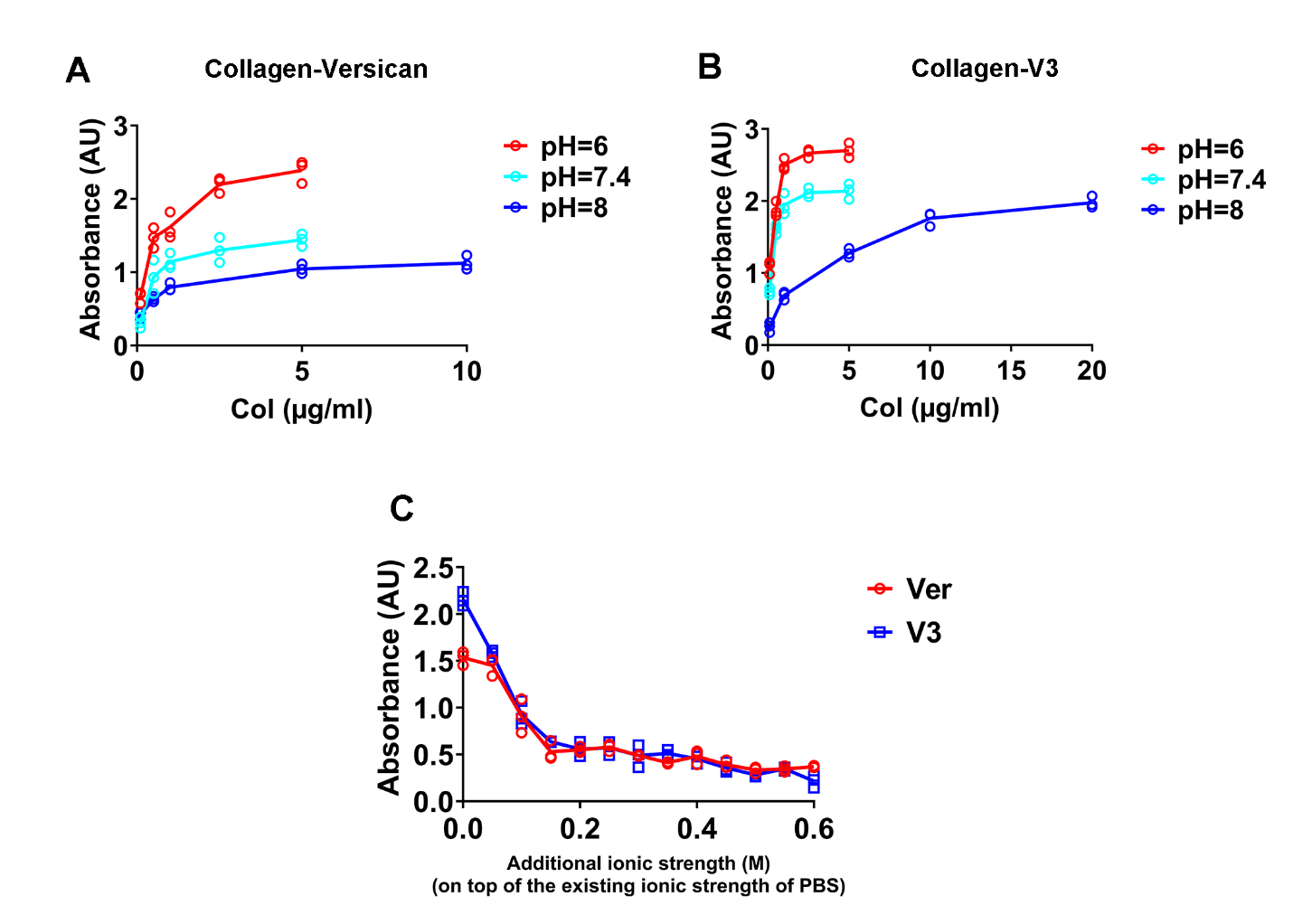


**Figure S2.** The interaction between collagen and versican is pH and ionic strength dependent. **A)** The effect of pH on collagen-versican interactions. **B)** The effect of pH on collagen-V3 interactions. **C)** The effect of ionic strength on collagen-versican and collagen-V3 interactions. The x-axis indicates the *additional* NaCl added to the binding buffer. Three independent experiments were carried out for each; all data were shown.


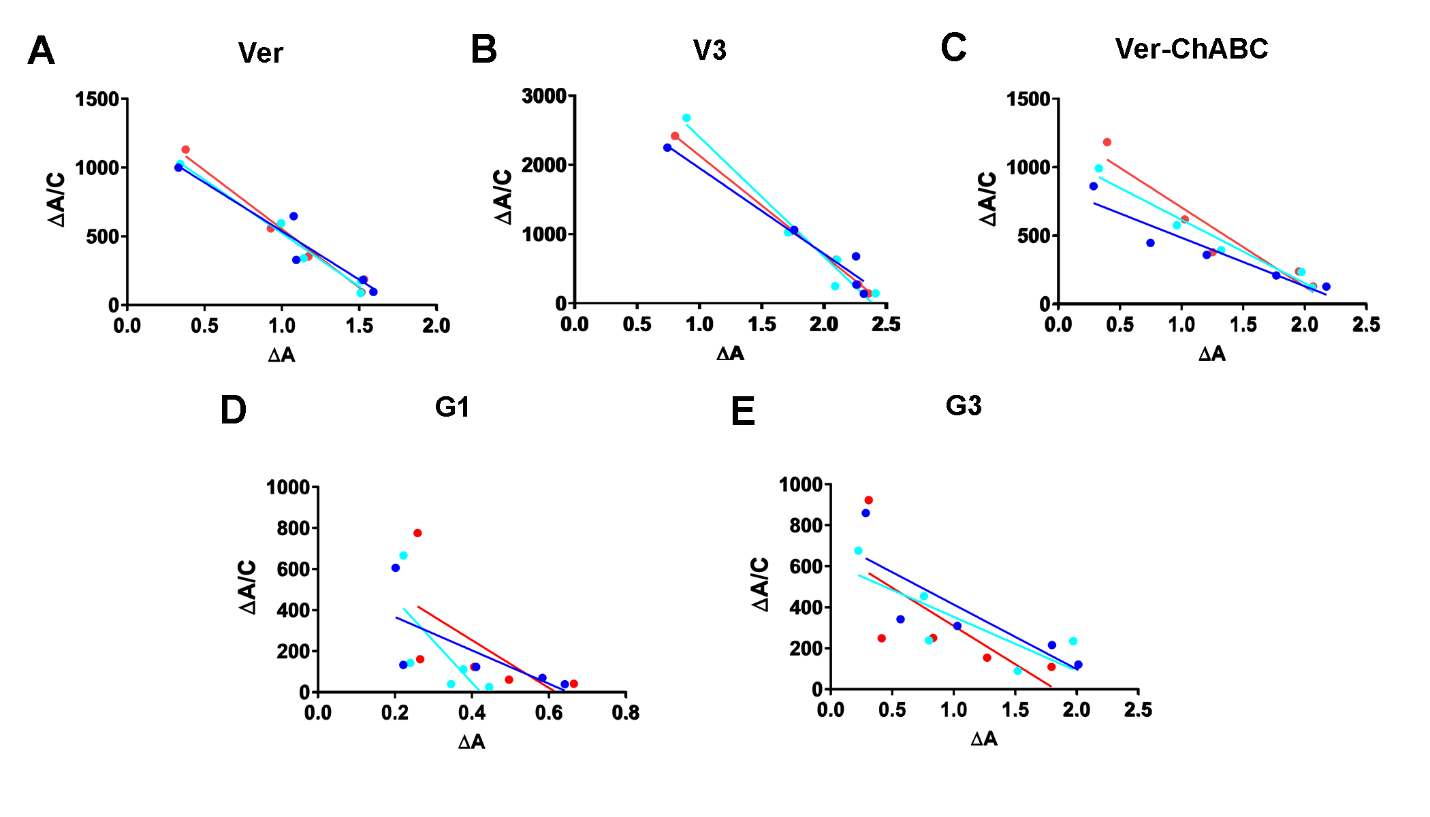


**Figure S3.** Scatchard analysis of solid phase binding data shown in Figure 2B & C. **A-E)** The Scatchard plot of the binding data from collagen-versican (A), collagen-V3 (B), collagen-versican digested with chondroitinase ABC (C), collagen-G1 domain (D) and collagen-G3 domain (E). Three independent experiments were carried out for each.


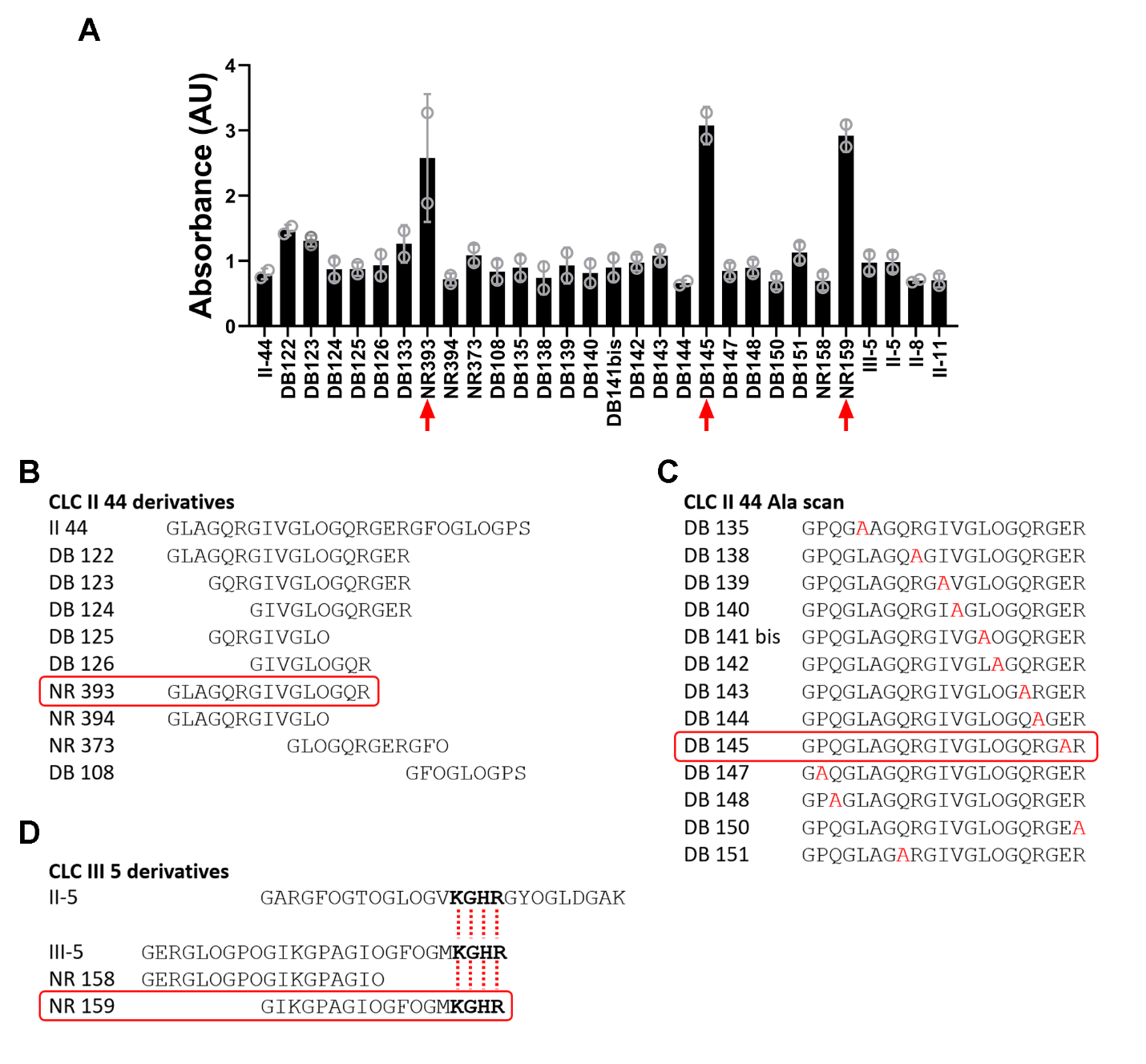


**Figure S4.** Versican G3 domain binds specific peptides with different motifs. **A)** Binding between variants of CLC II-44 and III-5 and 10 µg/ml recombinant G3 was tested using a solid-phase binding assay. **B)** The sequences of CLC II-44 derivatives. **C)** The sequences of CLC II-44 modified with alanine (Ala/A, red). **D)** The sequences of CLC III-5 derivatives. The high binding peptides are shown surrounded by a red box and the binding motifs (KGHR) found for peptide II-5, III-5 and III-5 variant are aligned with red dashed lines. Two independent experiments with three technical repeats each were carried out; data were adjusted by substracting the absorbance of the BSA control; each data point represents the mean of three technical repeats of each independent experiment; each bar represents mean ± SD.


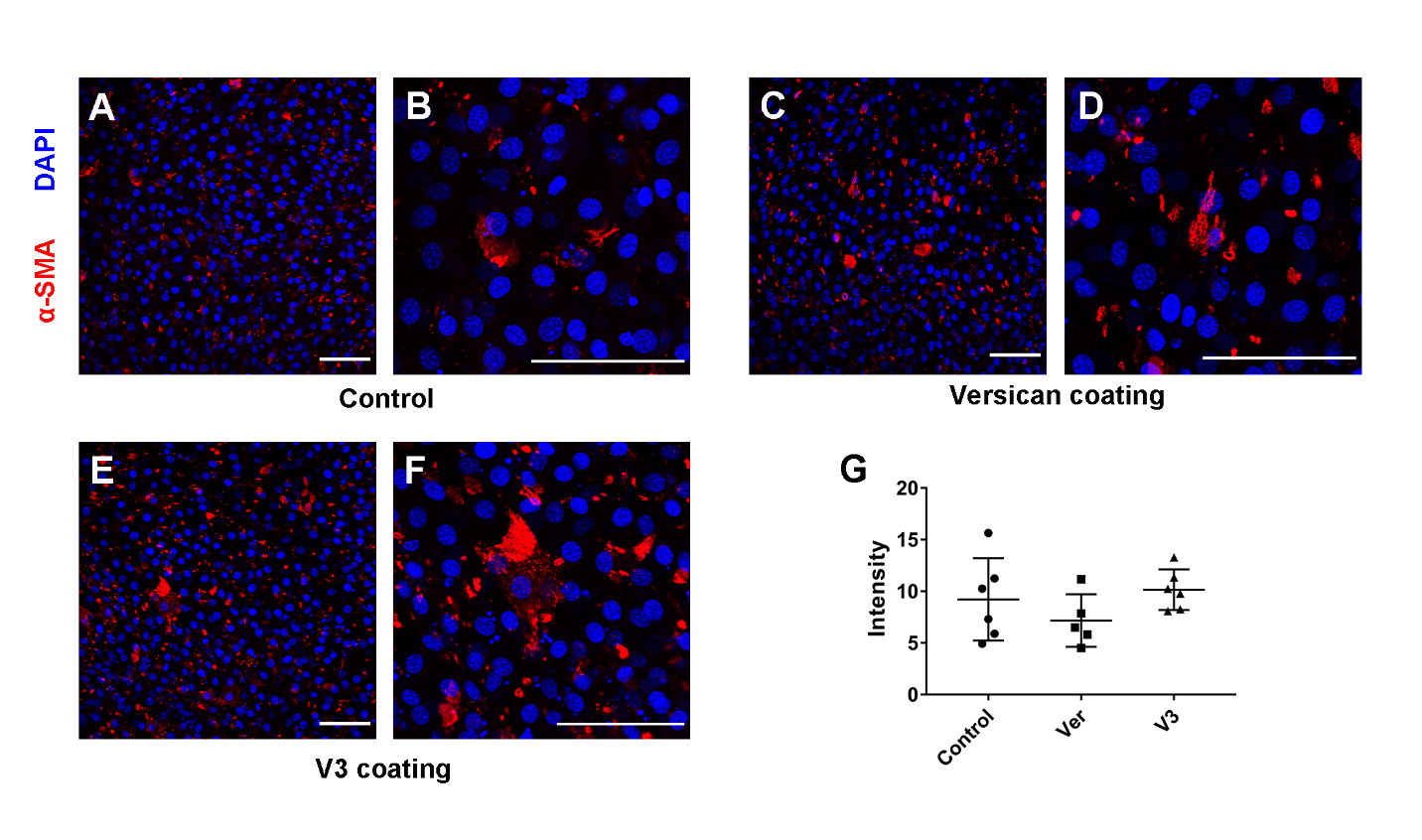


**Figure S5.** 3T3 fibroblasts on fibroblast-derived matrices are not activated due to the presence of versican or V3 isoform. **A-F)** Representative confocal imaging of fibroblast-derived matrices immunostained for α-smooth muscle actin (α-SMA): vitronectin coating on plate as a control (A, B); versican coating (C, D); V3 coating (E, F). Scale bar = 200 μm; note different magnifications of the panels in each pair; α-SMA (red), DAPI (blue). **G)** Quantification of α-SMA staining. One technical repeat from one individual experiment was used for α-SMA, and data points in (G) represent the intensity of α-SMA from images taken from each condition (6 images for control; 5 images for versican coating; 6 images for V3 coating). Data represent mean ± SD. No statistically significant difference in G between any pair of conditions was found, analyzed via one way ANOVA with Tukey’s multiple comparison.


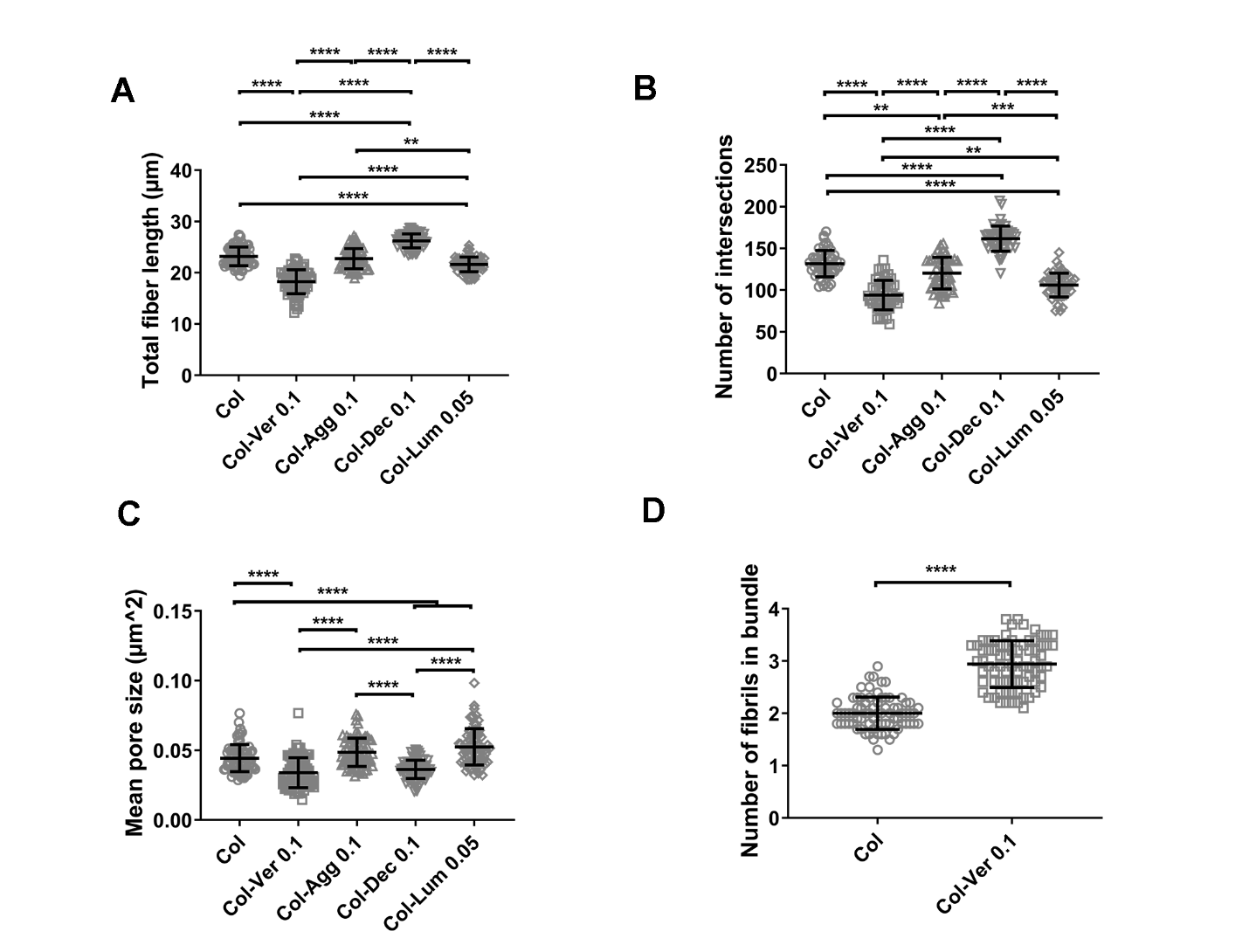


**Figure S6.** Versican and collagen form a loosely connected network with large fiber bundles and small pores. **A-C)** The total fiber length, number of intersections and mean pore size of collagen gels with different proteoglycans were quantified by DiameterJ using previously published SEM images (5). Versican (Ver), aggrecan (Agg) and decorin (Dec) were added at 0.1 mg/ml to 1.5 mg/ml collagen (Col)and lumican (Lum) was added at 0.05 mg/ml to 1.5 mg/ml collagen (Col). In A, p = 0.0014 for Col-Agg 0.1 vs. Col-Lum 0.05, p = 1.7e-6 for Col vs. Col-Lum 0.05 and p < 1.0e-15 for Col vs. Col-Ver 0.1, Col vs. Col-Dec 0.1, Col-Ver 0.1 vs. Col-Agg 0.1, Col-Ver 0.1 vs. Col-Dec 0.1, Col-Ver 0.1 vs. Col-Lum 0.05, Col-Agg 0.1 vs. Col-Dec 0.1, Col-Dec 0.1 vs. Col-Lum 0.05. In B, p = 0.0053 for Col vs. Col-Agg 0.1, p = 0.0031 for Col-Ver 0.1 vs. Col-Lum 0.05, p = 0.0002 for Col-Agg 0.1 vs. Col-Lum 0.05, p = 1.3e-12 for Col vs. Col-Lum 0.05, p = 4.2e-13 for Col-Ver 0.1 vs. Col-Agg 0.1 and p < 1.0e-15 for Col vs. Col-Ver 0.1, Col-Agg 0.1 vs. Col-Dec 0.1, Col-Dec 0.1 vs. Col-Lum 0.05, Col-Ver 0.1 vs. Col-Dec 0.1 and Col vs. Col-Dec 0.1. In C, p = 2.9e-7 for Col vs. Col-Ver 0.1, p = 5.7e-5 for Col vs. Col-Dec 0.1, p = 0.0045 for Col vs. Col-Lum 0.05, p = 8.2e-14 for Col-Ver 0.1 vs. Col-Agg 0.1, p < 1.0e-15 for Col-Ver 0.1 vs. Col-Lum 0.05, p = 1.5e-10 for Col-Agg 0.1 vs. Col-Dec 0.1 and p = 9.0e-15 for Col-Dec 0.1 vs. Col-Lum 0.05. **D)** The number of individual fibrils in bundles was counted manually from collagen and collagen-versican SEM images (p < 1.0 e-15). A and B were analyzed using one way ANOVA; C was analyzed using Kruskal-Wallis test with Dunn’s multiple comparison test; D was analyzed using unpaired t test; ** p < 0.01, *** p < 0.001 and **** p < 0.0001.


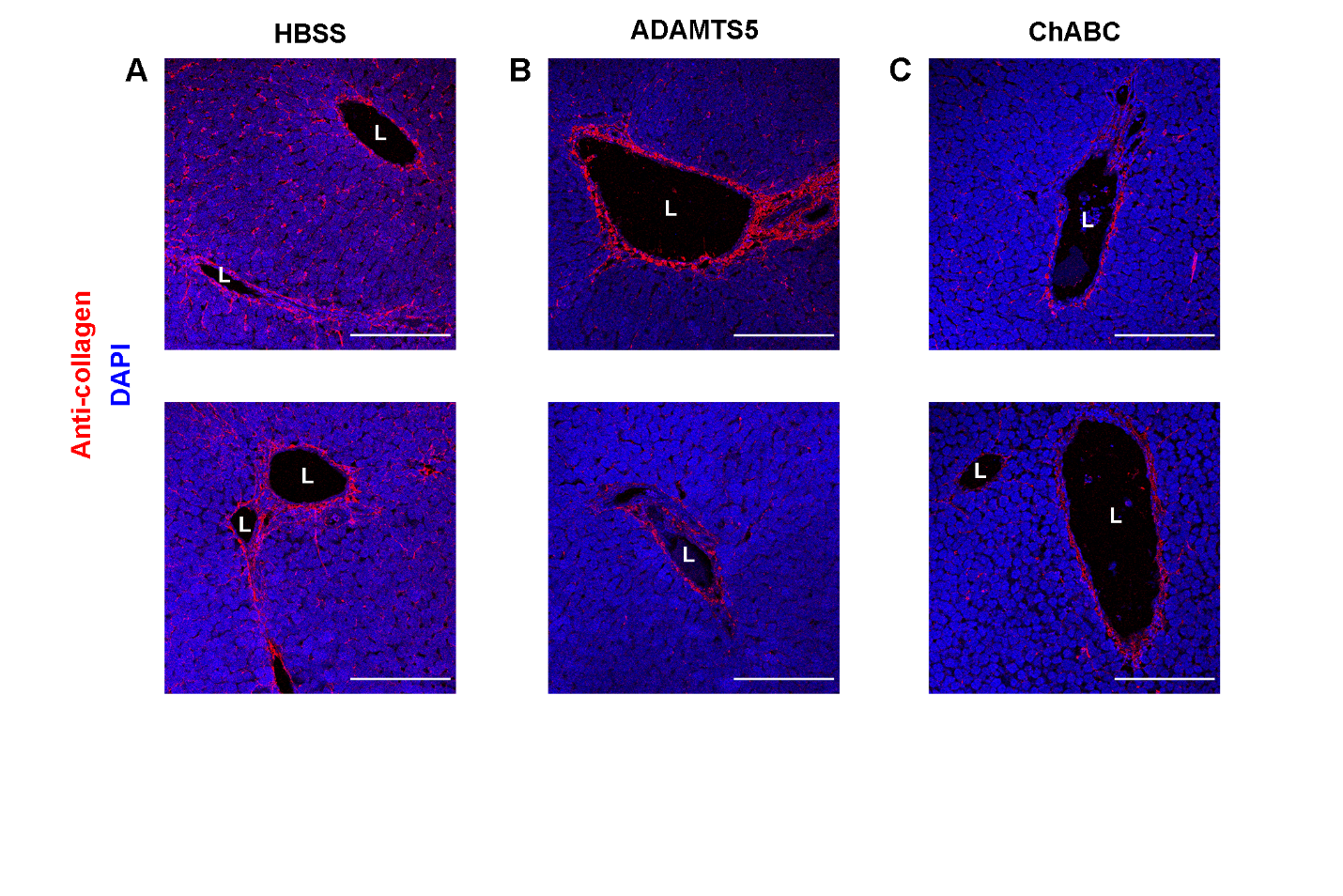


**Figure S7.** ADAMTS5 and chABC perfusions showed minimal impacts in collagen structure. **A-C)** Representative confocal images of collagen stained tissue in HBSS (A), ADAMTS5 (B) and ChABC (C) perfused livers. L indicates lumen of a portal tract or vessel. Scale bar = 200 μm. Anti-collagen staining (red), DAPI (blue).


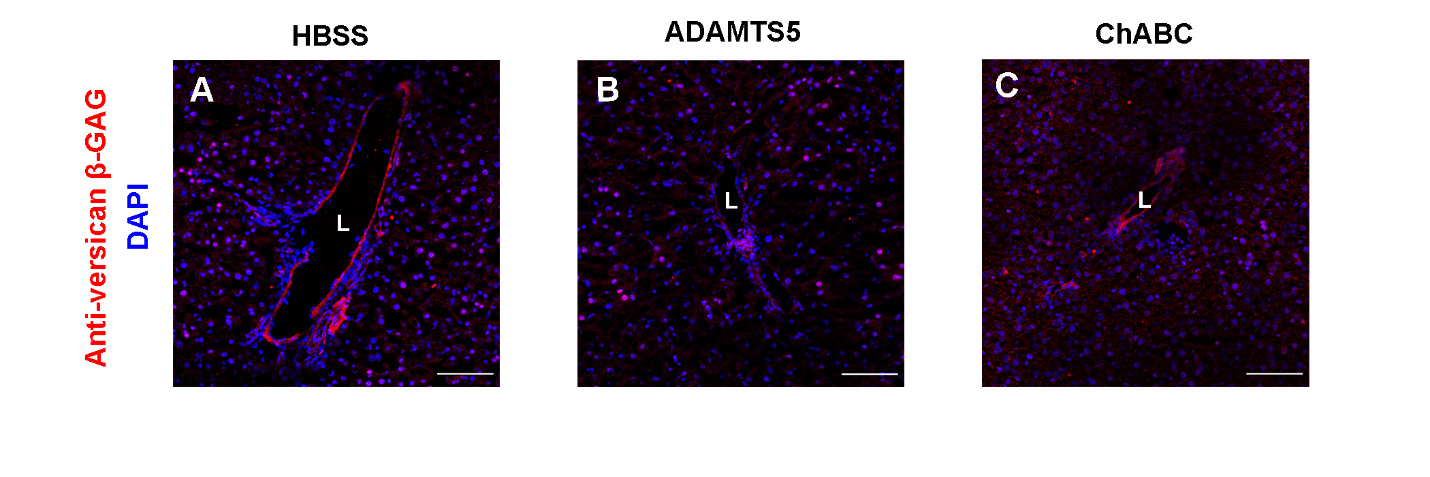


**Figure S8.** ADAMTS5 perfusion of liver tissues effectively cleaves versican core protein. **A-C)** Representative confocal images of versican β-GAG-stained tissue in HBSS (A), ADAMTS5 (B) and ChABC (C) perfused livers. L indicates lumen of a portal tract or vessel. Scale bar = 200 μm. Anti-versican staining (red), DAPI (blue). Anti-versican βGAG antibody targets amino acids 1360-1439 in the full-length protein, covering the ADAMTS5 cleavage site, and therefore only stains intact versican.

**Table S1.** The amino acid sequences and molecular weight of 56 Collagen Ligand Collection II peptides. O represents hydroxyproline.

| **Peptide** | **Sequence** | **MW** |
| --- | --- | --- |
| CLC-II-1 | GPC-(GPP)_5_-GPMGPMGPRGPOGPAGAOGPQGFQGNO-(GPP)_5_-GPC-NH_2_ | 5558 |
| CLC-II-2 | GPC-(GPP)_5_-GPQGFQGNOGEOGEOGVSGPMGPRGPO-(GPP)_5_-GPC-NH_2_ | 5648 |
| CLC-II-3 | GPC-(GPP)_5_-GPMGPRGPOGPOGKOGDDGEAGKOGKA-(GPP)_5_-GPC-NH_2_ | 5572 |
| CLC-II-4 | GPC-(GPP)_5_-GEAGKOGKAGERGPOGPQGARGFOGTO-(GPP)_5_-GPC-NH_2_ | 5621 |
| CLC-II-5 | GPC-(GPP)_5_-GARGFOGTOGLOGVKGHRGYOGLDGAK-(GPP)_5_-GPC-NH_2_ | 5710 |
| CLC-II-6 | GPC-(GPP)_5_-GYOGLDGAKGEAGAOGVKGESGSOGEN-(GPP)_5_-GPC-NH_2_ | 5533 |
| CLC-II-7 | GPC-(GPP)_5_-GESGSOGENGSOGPMGPRGLOGERGRT-(GPP)_5_-GPC-NH_2_ | 5668 |
| CLC-II-8 | GPC-(GPP)_5_-GLOGERGRTGPAGAAGARGNDGQOGPA-(GPP)_5_-GPC-NH_2_ | 5503 |
| CLC-II-9 | GPC-(GPP)_5_-GNDGQOGPAGPOGPVGPAGGOGFOGAO-(GPP)_5_-GPC-NH_2_ | 5385 |
| CLC-II-10 | GPC-(GPP)_5_-GGOGFOGAOGAKGEAGPTGARGPEGAQ-(GPP)_5_-GPC-NH_2_ | 5423 |
| CLC-II-11 | GPC-(GPP)_5_-GARGPEGAQGPRGEOGTOGSOGPAGAS-(GPP)_5_-GPC-NH_2_ | 5447 |
| CLC-II-12 | GPC-(GPP)_5_-GSOGPAGASGNOGTDGIOGAKGSAGAO-(GPP)_5_-GPC-NH_2_ | 5295 |
| CLC-II-13 | GPC-(GPP)_5_-GAKGSAGAOGIAGAOGFOGPRGPOGPQ-(GPP)_5_-GPC-NH_2_ | 5417 |
| CLC-II-14 | GPC-(GPP)_5_-GPRGPOGPQGATGPLGPKGQTGEOGIA-(GPP)_5_-GPC-NH_2_ | 5510 |
| CLC-II-15 | GPC-(GPP)_5_-GQTGEOGIAGFKGEQGPKGEOGPAGPQ-(GPP)_5_-GPC-NH_2_ | 5607 |
| CLC-II-16 | GPC-(GPP)_5_-GEOGPAGPQGAOGPAGEEGKRGARGEO-(GPP)_5_-GPC-NH_2_ | 5558 |
| CLC-II-17 | GPC-(GPP)_5_-GKRGARGEOGGVGPIGPOGERGAOGNR-(GPP)_5_-GPC-NH_2_ | 5628 |
| CLC-II-18 | GPC-(GPP)_5_-GERGAOGNRGFOGQDGLAGPKGAOGER-(GPP)_5_-GPC-NH_2_ | 5680 |
| CLC-II-19 | GPC-(GPP)_5_-GPKGAOGERGPSGLAGPKGANGDOGRO-(GPP)_5_-GPC-NH_2_ | 5529 |
| CLC-II-20 | GPC-(GPP)_5_-GANGDOGROGEOGLOGARGLTGROGDA-(GPP)_5_-GPC-NH_2_ | 5606 |
| CLC-II-21 | GPC-(GPP)_5_-GLTGROGDAGPQGKVGPSGAOGEDGRO-(GPP)_5_-GPC-NH_2_ | 5562 |
| CLC-II-22 | GPC-(GPP)_5_-GAOGEDGROGPOGPQGARGQOGVMGFO-(GPP)_5_-GPC-NH_2_ | 5650 |
| CLC-II-23 | GPC-(GPP)_5_-GQOGVMGFOGPKGANGEOGKAGEKGLO-(GPP)_5_-GPC-NH_2_ | 5625 |
| CLC-II-24 | GPC-(GPP)_5_-GKAGEKGLOGAOGLRGLOGKDGETGAA-(GPP)_5_-GPC-NH_2_ | 5536 |
| CLC-II-25 | GPC-(GPP)_5_-GKDGETGAAGPOGPAGPAGERGEQGAO-(GPP)_5_-GPC-NH_2_ | 5447 |
| CLC-II-26 | GPC-(GPP)_5_-GERGEQGAOGPSGFQGLOGPOGPOGEG-(GPP)_5_-GPC-NH_2_ | 5577 |
| CLC-II-27 | GPC-(GPP)_5_-GPOGPOGEGGKOGDQGVOGEAGAOGLV-(GPP)_5_-GPC-NH_2_ | 5458 |
| CLC-II-28 | GPC-(GPP)_5_-GEAGAOGLVGPRGERGFOGERGSOGAQ-(GPP)_5_-GPC-NH_2_ | 5638 |
| CLC-II-29 | GPC-(GPP)_5_-GERGSOGAQGLQGPRGLOGTOGTDGPK-(GPP)_5_-GPC-NH_2_ | 5917 |
| CLC-II-30 | GPC-(GPP)_5_-GTOGTDGPKGASGPAGPOGAQGPOGLQ-(GPP)_5_-GPC-NH_2_ | 5401 |
| CLC-II-31 | GPC-(GPP)_5_-GAQGPOGLQGMOGERGAAGIAGPKGDR-(GPP)_5_-GPC-NH_2_ | 5561 |
| CLC-II-32 | GPC-(GPP)_5_-GIAGPKGDRGDVGEKGPEGAOGKDGGR-(GPP)_5_-GPC-NH_2_ | 5525 |
| CLC-II-33 | GPC-(GPP)_5_-GAOGKDGGRGLTGPIGPOGPAGANGEK-(GPP)_5_-GPC-NH_2_ | 5444 |
| CLC-II-34 | GPC-(GPP)_5_-GPAGANGEKGEVGPOGPAGSAGARGAO-(GPP)_5_-GPC-NH_2_ | 5344 |
| CLC-II-35 | GPC-(GPP)_5_-GSAGARGAOGERGETGPOGPAGFAGPO-(GPP)_5_-GPC-NH_2_ | 5450 |
| CLC-II-36 | GPC-(GPP)_5_-GPAGFAGPOGADGQOGAKGEQGEAGQK-(GPP)_5_-GPC-NH_2_ | 5495 |
| CLC-II-37 | GPC-(GPP)_5_-GEQGEAGQKGDAGAOGPQGPSGAOGPQ-(GPP)_5_-GPC-NH_2_ | 5475 |
| CLC-II-38 | GPC-(GPP)_5_-GPSGAOGPQGPTGVTGPKGARGAQGPO-(GPP)_5_-GPC-NH_2_ | 5412 |
| CLC-II-39 | GPC-(GPP)_5_-GARGAQGPOGATGFOGAAGRVGPOGSN-(GPP)_5_-GPC-NH_2_ | 5436 |
| CLC-II-40 | GPC-(GPP)_5_-GRVGPOGSNGNOGPOGPOGPSGKDGPK-(GPP)_5_-GPC-NH_2_ | 5525 |
| CLC-II-41 | GPC-(GPP)_5_-GPSGKDGPKGARGDSGPOGRAGEOGLQ-(GPP)_5_-GPC-NH_2_ | 5561 |
| CLC-II-42 | GPC-(GPP)_5_-GRAGEOGLQGPAGPOGEKGEOGDDGPS-(GPP)_5_-GPC-NH_2_ | 5561 |
| CLC-II-43 | GPC-(GPP)_5_-GEOGDDGPSGAEGPOGPQGLAGQRGIV-(GPP)_5_-GPC-NH_2_ | 5531 |
| CLC-II-44 | GPC-(GPP)_5_-GLAGQRGIVGLOGQRGERGFOGLOGPS-(GPP)_5_-GPC-NH_2_ | 5705 |
| CLC-II-45 | GPC-(GPP)_5_-GFOGLOGPSGEOGKQGAOGASGDRGPO-(GPP)_5_-GPC-NH_2_ | 5551 |
| CLC-II-46 | GPC-(GPP)_5_-GASGDRGPOGPVGPOGLTGPAGEOGRE-(GPP)_5_-GPC-NH_2_ | 5514 |
| CLC-II-47 | GPC-(GPP)_5_-GPAGEOGREGSOGADGPOGRDGAAGVK-(GPP)_5_-GPC-NH_2_ | 5491 |
| CLC-II-48 | GPC-(GPP)_5_-GRDGAAGVKGDRGETGAVGAOGAOGPO-(GPP)_5_-GPC-NH_2_ | 5449 |
| CLC-II-49 | GPC-(GPP)_5_-GAOGAOGPOGSOGPAGPTGKQGDRGEA-(GPP)_5_-GPC-NH_2_ | 5431 |
| CLC-II-50 | GPC-(GPP)_5_-GKQGDRGEAGAQGPMGPSGPAGARGIQ-(GPP)_5_-GPC-NH_2_ | 5534 |
| CLC-II-51 | GPC-(GPP)_5_-GPAGARGIQGPQGPRGDKGEAGEOGER-(GPP)_5_-GPC-NH_2_ | 5644 |
| CLC-II-52 | GPC-(GPP)_5_-GEAGEOGERGLKGHRGFTGLQGLOGPO-(GPP)_5_-GPC-NH_2_ | 5746 |
| CLC-II-53 | GPC-(GPP)_5_-GLQGLOGPOGPSGDQGASGPAGPSGPR-(GPP)_5_-GPC-NH_2_ | 5427 |
| CLC-II-54 | GPC-(GPP)_5_-GPAGPSGPRGPOGPVGPSGKDGANGIO-(GPP)_5_-GPC-NH_2_ | 5409 |
| CLC-II-55 | GPC-(GPP)_5_-GKDGANGIOGPIGPOGPRGRSGETGPA-(GPP)_5_-GPC-NH_2_ | 5528 |
| CLC-II-56 | GPC-(GPP)_5_-GPRGRSGETGPAGPOGNOGPOGPOGPO-(GPP)_5_-GPC-NH_2_ | 5521 |

**Table S2.** Conservation of V3 binding sites in Type I collagen

| D-Period | D1 | D1 | D1 | D1 | D2 | D2 |
| --- | --- | --- | --- | --- | --- | --- |
| CLC peptide | II-1 | II-4 | II-8 | II-11 | II-15 | II-18 |
| α1[II] | RGPO | RGFO | RGRT | RGEO | KGEO | RGFO |
| α1[I] | RG**L**O | RG**L**O | RGR**O** | RGEO | KGEO | RGFO |
| α2[I] | RGPO | RGFO | RGR**V** | RGE**V** | KGEO | RG**L**O |

The Table shows the D-period location of the V3-binding Type II collagen CLC peptides and compares their sequence with the equivalent positions in the Type I collagen α-chains. Conserved but not identical residues are shown in **bold**.

**Table S3.**  Statistical significance of differences in fibril orientation between conditions. Data from Figure 4E were analyzed using two-way ANOVA with repeated measurements (post hoc test by Tukey’s multiple comparison test).

| **Data #1** | **Data #2** | **Angle range** | **Significance** |
| --- | --- | --- | --- |
| Control | Ver | -40° to -30° | 0.0053 |
| Control | Ver | -30° to -20° | 2.03e-7 |
| Control | Ver | -20° to -10° | 1.65e-8 |
| Control | Ver | -10° to 0° | 5.18e-8 |
| Control | Ver | 0° to 10° | 2.10e-5 |
| Control | Ver | 10° to 20° | 0.0002 |
| Control | Ver | 20° to 30° | 0.0005 |
| Control | Ver | 30° to 40° | 0.0064 |
| Control | V3 | -40° to -30° | 0.0412 |
| Control | V3 | -30° to -20° | 0.0201 |
| Control | V3 | -20° to -10° | 0.0298 |
| Control | V3 | 20° to 30° | 0.0066 |
| Control | V3 | 30° to 40° | 0.0010 |
| Control | V3 | 40° to 50° | 0.0061 |
| Control | V3 | 50° to 60° | 0.0309 |
| Ver | V3 | -30° to -20° | 0.0169 |
| Ver | V3 | -20° to -10° | 0.0003 |
| Ver | V3 | -10° to 0° | 0.0010 |
| Ver | V3 | 0° to 10° | 0.0027 |

**Table S4.** Statistical significance of differences in G’ for different collagen co-gels subjected to a strain sweep test. Data were analyzed using two-way ANOVA with repeated measurements (post hoc test by Tukey’s multiple comparison test).

| **Data #1** | **Data #2** | **Shear strain (%)** | **Significance** |
| --- | --- | --- | --- |
| Col | Col-Ver | 7.9433 | 0.0027 |
| Col | Col-Ver | 10 | 1.52e-6 |
| Col | Col-Ver | 12.5893 | 7.92e-12 |
| Col | Col-Ver | 15.8489 | 8.3e-14 |
| Col | Col-Ver | 19.9625 | 6.2e-14 |
| Col | Col-Ver | 25.1189 | 4.09e-12 |
| Col | Col-V3 | 1 | 0.0298 |
| Col | Col-V3 | 10 | 0.0396 |
| Col | Col-V3 | 12.5893 | 2.20e-8 |
| Col | Col-V3 | 15.8489 | 1.09e-13 |
| Col | Col-V3 | 19.9625 | 6.3e-14 |
| Col | Col-V3 | 25.1189 | 2.87e-11 |
| Col | Col-Agg | 15.8489 | 0.0065 |
| Col | Col-Agg | 19.9625 | 1.09e-13 |
| Col | Col-Agg | 25.1189 | 1.31e-6 |
| Col | Col-Dec | 19.9625 | 8.9e-14 |
| Col | Col-Dec | 25.1189 | 1.26e-8 |
| Col-Ver | Col-V3 | 7.9433 | 0.0181 |
| Col-Ver | Col-Agg | 7.9433 | 0.0217 |
| Col-Ver | Col-Agg | 10 | 1.75e-5 |
| Col-Ver | Col-Agg | 12.5893 | 5.99e-10 |
| Col-Ver | Col-Agg | 15.8489 | 1.26e-13 |
| Col-Ver | Col-Agg | 19.9625 | 3.71e-5 |
| Col-Ver | Col-Agg | 25.1189 | 0.0454 |
| Col-Ver | Col-Dec | 7.9433 | 0.0018 |
| Col-Ver | Col-Dec | 10 | 1.39e-7 |
| Col-Ver | Col-Dec | 12.5893 | 2.06e-13 |
| Col-Ver | Col-Dec | 15.8489 | 8.2e-14 |
| Col-V3 | Col-Dec | 10 | 0.0096 |
| Col-V3 | Col-Dec | 12.5893 | 4.15e-10 |
| Col-V3 | Col-Dec | 15.8489 | 1.08e-13 |
| Col-V3 | Col-Agg | 12.5893 | 1.55e-6 |
| Col-V3 | Col-Agg | 15.8489 | 1.36e-11 |
| Col-V3 | Col-Agg | 19.9625 | 0.0014 |
| Col-Agg | Col-Dec | 15.8489 | 0.0060 |
